# Neural network analysis as a novel skin outcome in a trial of belumosudil in patients with systemic sclerosis

**DOI:** 10.21203/rs.3.rs-4889334/v1

**Published:** 2024-10-15

**Authors:** Ilayda Gunes, Elana Bernstein, Shawn E. Cowper, Gauri Panse, Niki Pradhan, Lucy Duran Camacho, Nicolas Page, Elizabeth Bundschuh, Alyssa Williams, Mary Carns, Kathleen Aren, Sarah Fantus, Elizabeth R. Volkmann, Heather Bukiri, Chase Correia, Rui Wang, Vijaya Kolachalama, F. Perry Wilson, Seamus Mawe, J. Matthew Mahoney, Monique Hinchcliff

**Affiliations:** Yale School of Medicine, Department of Internal Medicine, Section of Rheumatology, Allergy, & Immunology; Columbia University; Yale School of Medicine, Departments of Dermatology and Pathology; Yale School of Medicine, Departments of Dermatology and Pathology; Columbia University; Yale School of Medicine, Department of Internal Medicine, Section of Rheumatology, Allergy, & Immunology; Yale School of Medicine, Department of Internal Medicine, Section of Rheumatology, Allergy, & Immunology; Yale School of Medicine, Department of Internal Medicine, Section of Rheumatology, Allergy, & Immunology; Yale School of Medicine, Department of Internal Medicine, Section of Rheumatology, Allergy, & Immunology; Northwestern University Feinberg School of Medicine, Department of Medicine, Division of Rheumatology; Northwestern University Feinberg School of Medicine, Department of Medicine, Division of Rheumatology; Northwestern University Feinberg School of Medicine, Department of Medicine, Division of Rheumatology; University of California, Los Angeles, David Geffen School of Medicine, Division of Rheumatology; University of California, Los Angeles, David Geffen School of Medicine, Division of Rheumatology; Northwestern University Feinberg School of Medicine, Department of Medicine, Division of Rheumatology; Sanofi, Translational medicine early development; Boston University Chobanian & Avedisian School of Medicine; Yale School of Medicine, Clinical and Translational Research Accelerator; Jackson Laboratory; Jackson Laboratory; Yale School of Medicine, Department of Internal Medicine, Section of Rheumatology, Allergy, & Immunology

**Keywords:** systemic sclerosis, scleroderma, modified Rodnan skin score, Deep Neural Network, AlexNet, belumosudil, outcome measure, outcomes, artificial intelligence, dermal fibrosis, skin fibrosis

## Abstract

**Background:**

The modified Rodnan skin score (mRSS), used to measure dermal thickness in patients with systemic sclerosis (SSc), is agnostic to inflammation and vasculopathy. Previously, we demonstrated the potential of neural network-based digital pathology applied to stained skin biopsies from SSc patients as a quantitative outcome. We leveraged deep learning and histologic analyses of clinical trial biopsies to decipher SSc skin features ‘seen’ by artificial intelligence (AI).

**Methods:**

Adults with diffuse cutaneous SSc (disease duration ≤ 6 years) enrolled in an open-label trial evaluating belumosudil underwent serial mRSS assessment and dorsal arm biopsies at week 0, 24 and 52/end of trial. Two blinded dermatopathologists independently scored stained sections [Masson’s trichrome, hematoxylin and eosin (H&E), CD3, CD34, CD8, α smooth muscle actin (αSMA)] for 16 published SSc dermal pathological parameters. We applied our previously published deep learning model to generate QIF signatures/biopsy and generated Fibrosis Scores. Associations between Fibrosis Score and mRSS (Spearman correlation); and between Fibrosis Score mRSS versus histologic parameters [odds ratios (OR)] were determined.

**Results:**

Only ten patients were enrolled because the sponsor terminated the trial early. Median, interquartile range (IQR) for mRSS change (0–52 weeks) for the five participants with paired biopsies was − 2.5 (−11—7.5), and for the ten participants was − 2 (−9—7.5). The correlation between Fibrosis Score and mRSS was R = 0.3; p = 0.674. Per 1-unit mRSS change (0–52W), histologic parameters with the greatest associated changes were (OR, p-value): telangiectasia (2.01, 0.001), perivascular CD3+ (1.03, 0.015), and % of CD8 + among CD3+ (1.08, 0.031). Likewise, per 1-unit Fibrosis Score change, parameters with greatest changes were (OR, p-value): hyalinized collagen (1.1, < 0.001), subcutaneous (SC) fat loss (1.47, < 0.001), thickened intima (1.21, 0.005), and eccrine entrapment (1.14, 0.046).

**Conclusions:**

Belumosudil was associated with a non-clinically meaningful improvement in mRSS. Fibrosis Score changes correlated with histologic feature changes (*e.g*., hyalinized collagen, SC fat loss) that were distinct from those associated with mRSS changes (*e.g*., telangiectasia, perivascular CD3+, and % of CD8 + among CD3+). These data suggest that AI applied to SSc biopsies may be useful for quantifying pathologic features of SSc beyond skin thickness.

## Background

Systemic sclerosis (SSc) is a rare chronic autoimmune disease whose pathogenesis involves fibrosis, inflammation, and vasculopathy including vascular pruning and thickened intima [1]. Systemic sclerosis subsets (limited cutaneous and diffuse cutaneous) are defined using the modified Rodnan skin score (mRSS), a semi-quantitative assessment of dermal thickness [(0 (normal) to 3 (hidebound) for 17 body sites (range of 0–51)] [2]. The score initially included assessment of 26 body areas, but nine (neck, upper back, lower back, and bilateral toes, shoulders and breasts) were dropped due to high inter-rater variation [3]. The construct validity of the mRSS is supported by the strong correlation (r = 0.81) between the forearm skin score and the dry weight of an adjacent 7-mm punch biopsies [3]. The mRSS is the current gold standard for assessing skin thickness in patients with SSc [4, 5]. However, despite promising pre-clinical data, results of clinical trials whose primary endpoint is the mRSS have been uniformly negative [6–21]. Perhaps, negative trial results are due to the mRSS being an incomplete readout of clinical changes as oppposed to ineffective therapies. Here, we demonstrate the potential utility of applying artificial intelligence (AI) to stained skin biopsy sections to measure comprehensively skin fibrosis, inflammation and vasculopathy in patients with early dcSSc enrolled in a belumosudil trial.

Artificial intelligence (AI) technology has been successfully applied in many fields including biomedical research and medical education to name a few [22, 23]. For example, AI has been shown to aid diagnosis, reduce medical errors, and improve medical education [24, 25]. We previously subjected forearm skin biopsies to AI methods for skin disease measurement in patients with SSc [26]. Briefly, we applied a pre-trained deep neural network (DNN AlexNet) to 100 randomly selected biopsy image patches (~ 0.16 mm^2^), to generate 4,096 quantitative image features (QIFs)/image patch. We used QIF signatures/biopsy to develop two regression models: 1) to predict whether a biopsy was from an SSc patient versus a healthy control (HC), and 2) to predict the mRSS (the output of this model is termed ‘Fibrosis Score’). The present research goal was to gain insights into the SSc histopathology features quantified by the DNN algorithm. We analyzed skin biopsies obtained during an open-label SSc trial of belumosudil. Belmudosudil, approved by the Food and Drug Administration in 2021 for chronic graft-versus-host-disease (cGVHD) treatment, is an inhibitor of Rho-associated coiled-coil containing protein kinase 2 (ROCK2) [27]. Belumosudil downregulates proinflammatory responses by inhibiting signal transducer and activator of transcription 3 (STAT3) phosphorylation, upregulating STAT5 phosphorylation, and shifting T helper 17 (Th17)/T regulatory (Treg) balance towards the Treg phenotype: all mechanisms that have been implicated in SSc skin disease [28].

We compared Fibrosis Scores to 16 previously published histological parameters associated with SSc, and to the mRSS [5]. Our findings show that our algorithm reproducibly quantifies features of SSc skin disease and quantifies skin features distinct from the mRSS semi-quantification of skin thickness. Moreover, we show that belumosudil may be an effective therapy in some patients with early dcSSc although larger clinical trials will be needed to assess efficacy.

## Methods

### Participants

The phase 2, open-label, belumosudil multicenter study protocol (Kadmon Corporation LLC., a Sanofi company, KD025–215) received Institutional Review Board approval at each participating site (Yale University, Columbia University, Northwestern University, and University of California, Los Angeles). Patient research partners provided written informed consent in accordance with the Declaration of Helsinki. Patients with SSc who fulfilled the 2013 American College of Rheumatology/European League Against Rheumatism Systemic Sclerosis Classification Criteria with SSc disease duration ≤ 6 years and diffuse cutaneous skin involvement (15 ≤ mRSS ≤ 40) were eligible [29, 30] (Table 1).

### Clinical assessment and dermal biopsies

Consenting participants received 200 mg belumosudil tablets twice daily for 52 weeks. Clinical data including mRSS and skin biopsies were obtained. Participants underwent 4mm dermal biopsies obtained from the non-dominant dorsal mid-forearm at baseline week 0, week 24, and week 52 and/or at end of trial (EOT). Biopsies were placed in formalin, transferred to 70% ethanol after 24h, and shipped to a central laboratory for analysis. Biopsies were paraffin-embedded, sectioned and stained with hematoxylin and eosin (H&E), Masson’s trichrome, CD3, CD8, CD31, CD34, and alpha- smooth muscle actin (αSMA) (all Leica Biosystems, Buffalo Grove, IL). The AlexNet DNN algorithm was applied to trichrome-stained biopsy sections.

Modified Rodnan skin score was assessed at weeks 0, 8, 16, 24, 36, and 52, and the primary trial outcome was the American College of Rheumatology (ACR)-endorsed Composite Response Index for Systemic Sclerosis (CRISS) response at week 24 [31, 32]. Briefly, the CRISS is a two-step process with assessment of newly impaired or worsening cardiac function (ejection fraction of ⩽45% requiring treatment), lung function (relative loss of forced vital capacity % predicted (FVC) of > 15 in patients with ILD) or new onset of pulmonary arterial hypertension (PAH), or the occurrence of scleroderma renal crisis during step one. In the absence of step one outcomes, five variables (FVC% predicted, mRSS, Physician Global Assessment (PGA), Patient Global Assessment (PtGA), and Health Assessment Questionnaire-Disability Index (HAQ-DI) are evaluated in step two, and the overall probability of improvement during the trial are reported [33].

### Histological analysis

**Figure 1:**
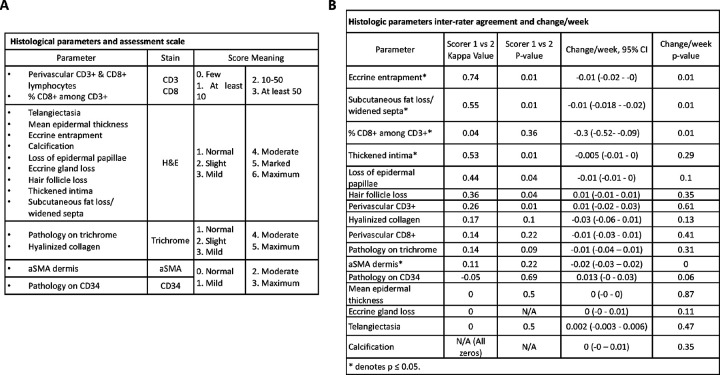
Systemic sclerosis dermatopathology parameters and scoring, rater agreement and parameter change. **A.** Scoring for 16 SSc dermatopathology parameters. **B.** Histologic parameter Kappa values and parameter score change per week (*indicates p-value<0.05).

Two blinded dermatopathologists independently scored slides for 16 histological parameters based upon previous SSc histopathology study results (Fig. 1a) [34–37]. Epidermal papilla loss, entrapment of at least one eccrine coil, complete loss of eccrine coil, presence of telangiectasia, loss of at least one hair follicle, presence of calcification, loss of subcutaneous fat or widening of the subcutaneous septa, presence of at least one thickened intima were scored as “yes” or “no.” Mean epidermal thickness was measured in micrometers (mm) at five randomly selected sites on the biopsy specimen, and the measurements were averaged. The H&E, trichrome, CD34, and αSMA staining parameters were scored numerically with reference to a standard set of images constructed by pathologist consensus (in advance) to optimize interobserver reproducibility. The range of scores in the standard image set extend from no staining to dense staining (Fig. 1a). To evaluate intraobserver reproducibility, each dermatopathologist reassessed histological features at two time points.

### Deep neural network feature extraction

**Figure 2:**
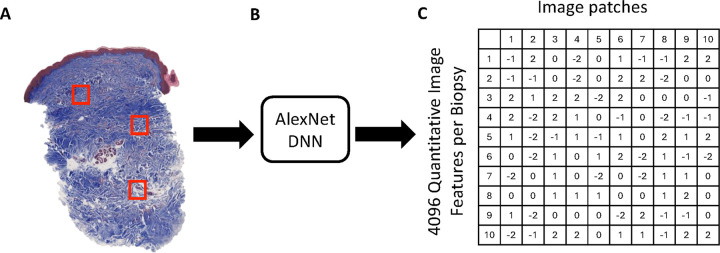
Deep Neural Network applied to trichrome-stained skin biopsy section. **A.** Image patches of skin biopsy section stained with trichrome. **B.** Artificial intelligence (AlexNet Deep Neural Network) applied to each image patch (100 per biopsy). **C.**Quantitative Image Features generated for each image patch.

Skin biopsy sections were imaged using a Hamamatsu Nanozoomer S210 whole-slide scanner (Shizuoka, Japan) at 40x objective, and images were saved as .ndpi files. Because the .ndpi file format is designed for storage, these images were converted into Zarr files for computational investigation. Zarr files were transformed into QIFs using the AlexNet DNN model, pretrained on ImageNet, available from the ONNX Model Zoo [38]. The AlexNet algorithm was applied to each of 100 randomly selected image patches (~ 0.16 mm^2^) from the dermis (epidermis and subcutis were excluded) to generate a new set of 4,096 QIFs for each image patch (100 × 4,096 QIF matrix) (Fig. 2).

### Linear regression model and association with mRSS and local skin score

As in prior work, we developed a linear regression model comprised of QIF as predictor variables and the mRSS as the outcome variable (model output termed ‘Fibrosis Score’). We selected lambda from a grid of 16 values logarithmically spaced between 10^− 5^ (weak penalty) and 10^3^ (strong penalty). For each image patch and value of lambda, the linear model predicted a value for mRSS as a linear combination of the QIF levels plus an intercept (which accounts for the mean mRSS of the training population). To integrate from image patches to biopsies, we averaged the predicted scores for the 100 image patches within a biopsy to obtain a Fibrosis Score. The model was cross validated by holding out sets of patients so that we avoided within-patient correlations.

### Statistical Analyses

Inter- and intra-rater variability for 16 histological assessments were calculated using Cohen’s Kappa values [39]. Kappa values ≤ 0 = no agreement, 0.01–0.20 = none to slight, 0.21–0.40 fair, 0.41– 0.60 = moderate, 0.61–0.80 substantial, and 0.81–1.00 = almost perfect agreement [39]. Ordinal logistic regression models were used to calculate odds ratios (OR), the relationship between an ordinal response variable (the mRSS and the Fibrosis Score, separately) and one or more explanatory variables (histological parameters). The dermatopathologists’ parameter score change/week average from their two timepoints was computed by fitting each measurement into a mixed effects model, with random effects for participant and slope and an independent covariance structure. Spearmen’s rank correlation coefficient was used to measure correlations between mRSS and the Fibrosis Score. As mentioned above, a lasso regression model trained using a previously validated data set to read trichrome-stained biopsy skin sections was applied to obtain a ‘Fibrosis Score’ (arbitrary range approximating the mRSS range 0–51) [26]. The Fibrosis Score was compared to mRSS through a correlation plot and to histological parameters through an ordinal logistic regression model. A p-value < 0.05 was considered statistically significant. All statistical analyses were obtained using Stata, version 18 (Statacorp, College Station, TX). No corrections for multiple hypothesis testing were made due to the exploratory nature of this study.

## Results

### Participant Data

**Figure 3:**
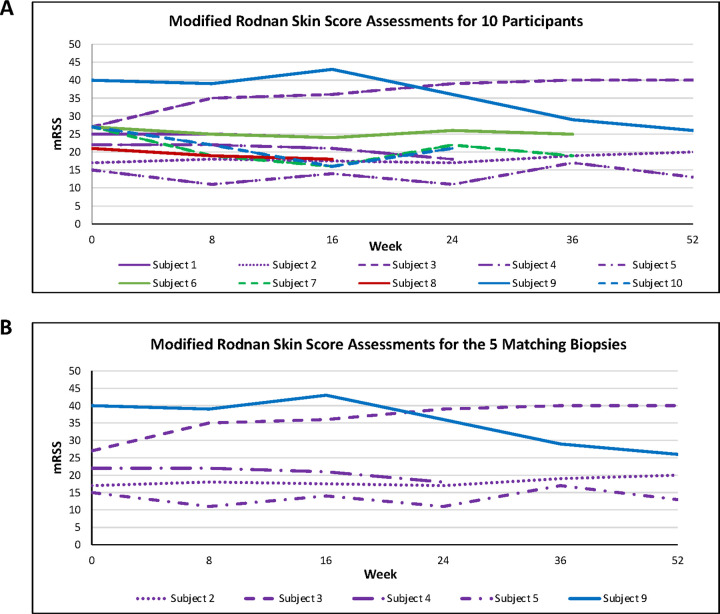
Modified Rodnan skin score trajectories. **A.** Ten participants’ mRSS W0 to last follow-up. The median interquartile range (IQR) mRSS change 0–52W was −2.5 (−11—7.5). **B.**Five participants’, with matching biopsies, mRSS W0 to last follow-up. The median interquartile range (IQR) mRSS change 0–52W was −2 (−9—7.5).

**Figure 4:**
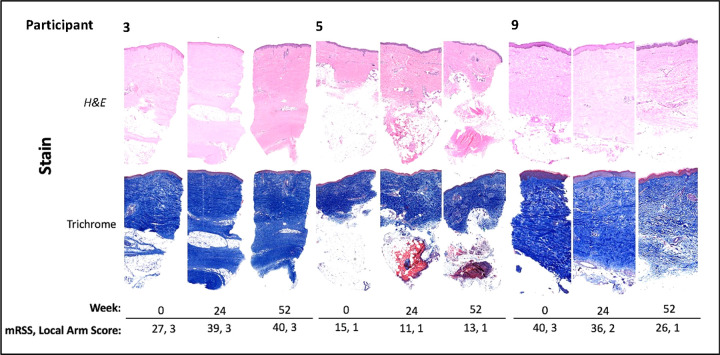
Stained sections for participants with 0-, 24- and 52-week biopsies. H&E and trichrome-stained biopsies for three participants during treatment with their mRSS and local arm scores.

Ten participants were recruited for this pilot study designed to test preliminarily the safety of belumosudil for the treatment of skin fibrosis in patients with early dcSSc. All ten recruited patients were women, eight were White (Non- Hispanic) and two were Black (Non- Hispanic). Participants had a mean (SD) age of 49.6 (7.3) years and dcSSc duration of 4.46 (1.01) years, respectively. The median interquartile range (IQR) mRSS change from 0–52W was − 2.5 (−11—7.5) (Fig. 3a). For the five participants with paired biopsies, week 0 and 24, the median (IQR) mRSS change 0–52W was − 2 (−9—7.5) (Fig. 3b). Although skin biopsy kits were sourced from a contracted research organization, tissue fixation issues at each participating site resulted in biopsies from only five out of ten patients suitable for analysis (Fig. 4).

### Histological Analysis

Eccrine entrapment (substantial agreement), subcutaneous fat loss/widened septum (moderate agreement), thickened intima (moderate agreement), and loss of epidermal papillae (moderate agreement) were the histological parameters with best inter-rater agreement [39] (Fig. 1b). Out of the 16 SSc parameters, significant change per week (from baseline to 52w) was observed for eccrine entrapment, subcutaneous fat loss/widened septum, and % CD 8 + among CD3 + lymphocytes (Fig. 1b).

### DNN-Fibrosis Score and mRSS

**Figure 5:**
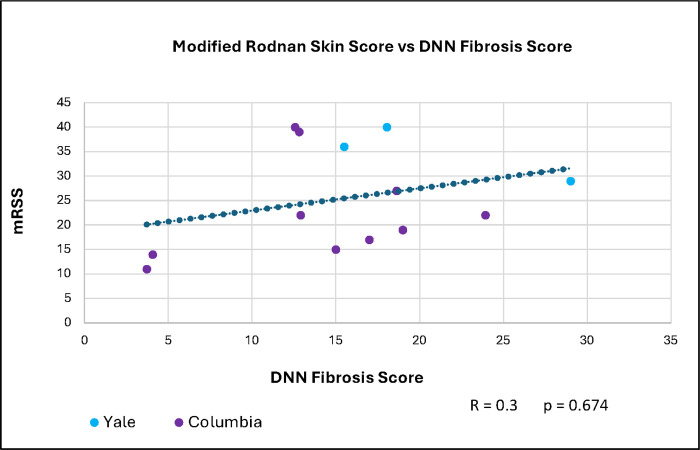
Correlation between modified Rodnan skin score (mRSS) and DNN Fibrosis Score. The Spearman correlation between mRSS and DNN Fibrosis Scores for five participants with baseline and follow-up biopsies.

The correlation between the five participants’ mRSS for all time points and DNN generated Fibrosis Scores for all biopsies was R = 0.3 and p = 0.674 (Fig. 5).

### Relationship between mRSS and Fibrosis Score and the histological parameters

Per 1-unit mRSS change, the histological parameters with significant associated changes (OR, p-value) were: telangiectasia = 2.01 (p = 0.001); perivascular CD3 + lymphocytes = 1.14 (p = 0.015); and % of CD8 + among CD3 + cell = 1.03 (p = 0.031). Similarly, per 1-unit Fibrosis Score increase, the histological parameters with significant associated changes (OR, p-value) were: subcutaneous fat loss/widened septum = 1.47 (p = 0.00033); thickened intima = 1.21 (p = 0.005); eccrine entrapment = 1.14 (p = 0.046), and hyalinized collagen = 1.1 (p = 0.00033) (Table S1).

## Discussion

The mRSS, developed in the 1970s, remains the gold standard skin thickness assessment used in SSc clinical trials [3]. In spite of its inclusion as one of five components of the revised CRISS [4, 32], the mRSS has several limitations: it is only semi-quantitative, only assesses dermal thickness, requires long intervals between repeated measurements to observe clinically meaningful changes, and is confounded by obesity and edema [40]. Identification of a new SSc skin outcome that is quantitative, reproducible, sensitive to early changes, and inclusive of the three pathologic SSc features (fibrosis, inflammation and vasculopathy) would likely improve our ability to identify effective treatment for SSc skin disease. We used skin biopsies from a clinical trial to, 1) test the potential feasibility and utility of the DNN-derived Fibrosis Score as an SSc skin outcome, and 2) determine the histologic features (using published SSc histological features) that the DNN algorithm “sees” and quantifies. We found that, 1) different histological parameters were significantly associated with mRSS versus Fibrosis Score changes, and 2) a weak correlation between the mRSS and DNN Fibrosis Score. Both results suggest that the DNN Fibrosis Score quantifies more than just skin thickness.

Alternative approaches for quantifying SSc skin disease have been explored including durometry [41], optical coherence tomography [42], and histological readouts (hyalinized collagen, pathology on trichrome stain and loss of dermal papillae) [37]. Early dermatohistopathological descriptions of SSc skin include a 1957 paper where researchers showed the pathological features of a variety of clinical presentations of SSc to be indistinguishable. They reported: 1) epidermal (epidermal hyperkeratosis/increased pigmentation of the basal epidermal layer, and atrophy and flattening of the rete ridges); 2) dermal (collagen hyalinization/homogenization, increased collagen fibril size with increased interbundle and cutis size, and collagen fibers with vacuolization); 3) vascular [thickened blood vessel walls (media and intima in skin and muscle), endothelial cell swelling, dilation of superficial capillaries, perivascular infiltrates], and 4) adnexal structure (sweat gland and duct atrophy, and hair follicle and sebaceous gland loss) were SSc features that were consistent across presentations [36]. Results of a 2006 study suggested that myofibroblast number and hyalinized collagen alterations are relevant in SSc skin disease [35] while results of a 2009 study suggested that myofibroblast number, narrowing of the arteriolar lumen in the deep vascular plexus (reticular dermis) and decreased dermal vascular density were significantly associated with increased skin thickness [34]. Based upon these three studies, combined with the results of a 2011 study that presented 14 parameters that were significantly associated with local skin thickness [37], our two SSc expert dermatopathologists assessed 16 parameters: epidermal thickness, density of perivascular CD3 + and CD8 + lymphocytes, % CD8 + among CD3 + lymphocytes, presence of calcification, and presence of subcutaneous fat loss/widened septa. In addition, overall matrix appearance (H&E and trichrome stains), and CD34 and alpha-smooth muscle actin immunohistochemical staining (assessed by direct comparison to a standardized image set). Parakeratosis, pigment incontinence, and mean epidermal pigmentation were excluded from analysis because they are confounded by skin color and degree of external manipulation. We also excluded focal lymphocyte exocytosis and increased mucin deposition in the deep dermis because they were not evident in any of the examined specimens.

The parameters with best inter-rater consistency were eccrine entrapment, subcutaneous fat loss/widened septum, thickened intima, and loss of epidermal papillae. Parameters with the worst agreement were pathology on CD34 staining, mean epidermal thickness, eccrine gland loss, and telangiectasia. Our results are consistent with histopathology results in other diseases where agreement among experts can be variable underscoring the need for assistive devices like AI [43].

Artificial intelligence is gaining traction in medicine. For instance, a 2020 paper that examined the performance of an AI system for prostate cancer detection (trained using 6953 prostate biopsy slides) compared to gold standard international urological pathologists’ review showed that the area under the curve was 0.997 (95% CI 0.994–0.000) for an independent test dataset (1631 prostate biopsy slides) [43]. Since SSc, unlike prostate cancer, is a rare disease, collaboration will be critical to ensure sufficient SSc patient biopsies (accounting for varying degrees of skin pigmentation and skin fibrosis) for training a useful AI model. Artificial intelligence tools could help improve diagnostic accuracy to facilitate early treatment initiation [44] and to assess early treatment response to shorten SSc clinical trial length from twelve months. Another application would be image analysis of skin biopsies to identify predictive and severity biomarkers of pulmonary arterial hypertension (PAH), interstitial lung disease (ILD), calcinosis cutis, and digital ulcers. Identifying patients at higher risk for these SSc-associated complications will help to inform the development of more rationale screening and treatment protocols.

The ability to apply AI (such as our DNN model) to skin biopsies as a novel SSc skin outcome would improve clinical care and clinical trial design. Skin biopsies can readily be performed at medical centers around the globe, fixed in formalin and shipped to a central analysis site. This would drastically increase the number of recruitment sites and the number of potential clinical trial participants. This should also ensure a diverse population of trial participants so trial results are more generalizable. We learned that supplies from contracted research organizations should be tested for quality assurance. Only 50% of the biopsies obtained in our study could be analyzed due to tissue slide adherence problems traced back to fixation. If rapid collection and analysis can be guaranteed, ethanol stabilization could be eliminated as a fixation step, thus minimizing the complexity of tissue fixation troubleshooting. An AI SSc skin outcome would permit reanalysis of archived biopsies from clinical trials to determine if a subset of patients had responded. It is possible that some abandoned SSc treatments may have benefitted a patient subset on the histopathological level as assessed by AI. Moreover, AI approaches in SSc skin disease would provide a quantitative and reproducible outcome and might shorten clinical trial duration due to earlier detection of small, but significant, changes. Lastly, AI algorithms can be trained to quantify inflammation, vasculopathy, and increased dermal thickness though quantifying the absence of a feature (e.g., loss of eccrine glands or hair follicles) will likely be more challenging.

We analyzed forearm skin biopsies because we, and others, have shown that forearm skin score (0–3) strongly and significantly correlates with mRSS [26, 45]. Thus, a quantitative AI-generated score of forearm skin disease could be a surrogate for total body skin disease. We view the low correlation between mRSS and Fibrosis Scores as a study strength because it suggests that our AI-generated score quantifies skin features beyond skin thickness. This study’s small sample size precludes drawing firm conclusions but does provide an important proof-of-concept: clinical trials can be designed to include AI-generated skin outcomes as a complement to the mRSS. Of course, with any AI assessment of stained skin biopsies, staining batch effects must be addressed and overcome. This can be accomplished with larger sample sizes and inclusion of additional stains.

Eight of the ten participants demonstrated mRSS improvement between baseline and last assessment, but only four patients demonstrated clinically significant changes as defined as at least 20% or 25% improvement from baseline score [31, 32], and all demonstrated RNA polymerase III serum antibodies. Two of these four patients had a mRSS reduction of greater than five points, thus supporting consideration of a larger, randomized, placebo-controlled trial of belumosudil.

We compared the histologic features that were significantly associated with the DNN-generated Fibrosis Score with those that were significantly associated with mRSS to assess concordance. Importantly, we found that mRSS improvement mirrored decreases in telangiectasia, perivascular CD3+, and % CD8 + among CD3 + cells, while Fibrosis Score changes were associated with hyalinized collagen, subcutaneous fat loss, thickened intima, and eccrine entrapment during belumosudil treatment. These findings indicate that the mRSS and DNN-derived Fibrosis Score measure distinct pathological features, and that combining the two approaches may be better than using either one in isolation. Thus, in addition to patient-reported outcome measures that assess treatment-associated feel and function changes, the best use of the Fibrosis Score would be inclusion as a complementary outcome to the mRSS.

Study limitations include the small dataset of only five participants with longitudinal biopsies. Reasons for this include early study withdrawal for two patients due to perceived lack of efficacy, and the death of one patient who died of causes deemed unrelated to the study drug. Another manuscript will be published that specifically reports detailed clinical trial results. Other reasons include early study termination before planned enrollment was completed and tissue fixation issues that resulted in 50% unusable biopsies. We attribute this to faulty skin biopsy collection kits because unusable biopsies were obtained from each of the four participating sites. Study strengths include our multicenter study design, our application of a published AI approach for quantifying skin disease, and the use of real-world clinical trial biopsies. The comprehensive histologic assessments for 16 skin features by two dermatopathologists at two time points is an additional strength. We acknowledge that our previously published DNN model, utilized in this study, is based on a pre-trained neural network originally trained on natural images. Future iterations of our modeling framework will aim to incorporate more advanced techniques to fully capture the information within histological images. This may include developing more sophisticated frameworks such as graph neural networks [46–48] and improving averaging methods across selected patches to better represent the comprehensive details of the histological images.

With the growing number of proposed therapies for SSc skin disease, there is an urgent need to increase the number of investigative sites that can participate in trials. We have shown that DNN analysis of skin biopsies is a feasible SSc skin outcome for use in clinical trials that is quantitative and comprehensive. Currently, we are analyzing archived biopsies from SSc patients obtained at other institutions, working to determine the optimal stain(s) (*e.g.*, CD3, CD8 and CD34) for use with AI, and annotating hundreds of slides for dozens of SSc features to permit us to train additional AI models with enhanced performance.

## Conclusions

To develop a quantitative SSc skin outcome that assesses skin thickness, inflammation and vasculopathy, we applied our previously published DNN algorithm to stained skin biopsies obtained during a clinical trial of belumosudil in patients with early dcSSc. We examined the relationship between the DNN-generated Fibrosis Score, mRSS, and important SSc histological parameters. The distinction between histologic parameters significantly associated with Fibrosis Score and mRSS suggests that the DNN algorithm may be a useful strategy for quantifying SSc pathologic features involved in SSc skin disease separate from dermal fibrosis. Ongoing work includes analyses of larger cohorts and training the DNN algorithm with *H&E*-, in addition to trichrome-, stained samples. The potential impact of integrating AI into the analysis of SSc skin biopsy images can facilitate the implementation of comprehensive, reproducible outcome measures, thereby streamlining clinical trials, transforming the pace of global recruitment, and increasing diversity and thus generalizability of SSc clinical trials.

## Figures and Tables

**Table 1:** Baseline study participant data

Characteristic mean (SD) or as indicated	
**Age**	49.6 (7.3)
**dcSSc duration (y)**	4.46 (1.01)
**Female, n (%)**	10 (100)
**Race/Ethnicity, n (%)**	
White, Non-Hispanic	8 (80)
Black or African American, Non-Hispanic	2 (20)
**Participant Baseline Clinical Data**	
Positive Anti-Scl-70, n (%)	1 (12.5)
Positive Anti-RNA polymerase III, n (%)	2 (25)
Baseline FVC	85 (20)
Baseline DLCO	75 (14)
ILD on HRCT	1 (11)

dcSSc = FVC = forced vital capacity % predicted, DLCO = diffusion capacity for carbon monoxide % predicted, ILD = interstitial lung disease, HRCT = chest high-resolution computed tomography

## Abbreviation Definition

SSc: Systemic sclerosis
lcSSc: Limited cutaneous systemic sclerosis
dcSSc: Diffuse cutaneous systemic sclerosis
mRSS: Modified rodnan skin score
DNN: Deep neural network
QIFs: Quantitative image features
W: Week
EOT: End of trial
SC: Subcutaneous
AI: Artificial intelligence
ACR: American College of Rheumatology
CRISS: Composite Response Index in Systemic Sclerosis
FVC: Forced vital capacity
ILD: Interstitial lung disease
PAH: Pulmonary arterial hypertension
H&E: Hematoxylin and eosin
aSMA: α-Smooth muscle actin
IQR: Interquartile range
PGA: Physician global assessment
PtGA: Patient global assessment
cGVHD: Chronic graft-versus-host disease
ROCK2: Rho-associated coiled-coil containing protein kinase 2
STAT: Signal transducer and activator of transcription
